# Collaborative care for depression and anxiety disorders: results and lessons learned from the Danish cluster-randomized Collabri trials

**DOI:** 10.1186/s12875-020-01299-3

**Published:** 2020-11-18

**Authors:** Nadja Kehler Curth, Ursula Ødum Brinck-Claussen, Carsten Hjorthøj, Annette Sofie Davidsen, John Hagel Mikkelsen, Marianne Engelbrecht Lau, Merete Lundsteen, Claudio Csillag, Kaj Sparle Christensen, Marie Jakobsen, Anders Bo Bojesen, Merete Nordentoft, Lene Falgaard Eplov

**Affiliations:** 1 Copenhagen Research Center for Mental Health – CORE, Mental Health Center Copenhagen, Mental Health Services, Gentofte Hospitalsvej 15, 2900 Hellerup, Denmark; 2 Department of Public Health, Section of Epidemiology, University of Copenhagen, Copenhagen, Denmark; 3 The Research Unit for General Practice and Section of General Practice, University of Copenhagen, Øster Farimagsgade 5, Postbox 2099, 1014 Copenhagen K, Denmark; 4 Mental Health Center Frederiksberg, Mental Health Services, Nordre Fasanvej 57-59, 2000 Frederiksberg, Denmark; 5 Stolpegård Psychotherapy Center, Mental Health Services, Stolpegårdsvej 20, 2820 Gentofte, Denmark; 6 General Practitioner, Copenhagen, Denmark; 7 Mental Health Center North Zealand, Mental Health Services, Dyrehavevej 48, 3400 Hillerød, Denmark; 8 Department of Public Health, Aarhus University, Aarhus, Denmark; 9 Research Unit for General Practice, Institute of Public Health, Aarhus University, Bartholins Allé 2, 8000 Aarhus C, Denmark; 10 VIVE – The Danish Center for Social Science Research, Herluf Trolles Gade 11, 1052 Copenhagen K, Denmark; 11 Institute for Clinical Medicine, University of Copenhagen, Copenhagen, Denmark

**Keywords:** Collaborative care, Anxiety disorders, Depression, General practice, Primary health care

## Abstract

**Background:**

Meta-analyses suggest that collaborative care (CC) improves symptoms of depression and anxiety. In CC, a care manager collaborates with a general practitioner (GP) to provide evidence-based care. Most CC research is from the US, focusing on depression. As research results may not transfer to other settings, we developed and tested a Danish CC-model (the Collabri-model) for depression, panic disorder, generalized anxiety disorder, and social anxiety disorder in general practice.

**Methods:**

Four cluster-randomized superiority trials evaluated the effects of CC. The overall aim was to explore if CC significantly improved depression and anxiety symptoms compared to treatment-as-usual at 6-months’ follow-up. The Collabri-model was founded on a multi-professional collaboration between a team of mental-health specialists (psychiatrists and care managers) and GPs. In collaboration with GPs, care managers provided treatment according to a structured plan, including regular reassessments and follow-up. Treatment modalities (cognitive behavioral therapy, psychoeducation, and medication) were offered based on stepped care algorithms. Face-to-face meetings between GPs and care managers took place regularly, and a psychiatrist provided supervision. The control group received treatment-as-usual. Primary outcomes were symptoms of depression (BDI-II) and anxiety (BAI) at 6-months’ follow-up. The incremental cost-effectiveness ratio (ICER) was estimated based on 6-months’ follow-up.

**Results:**

Despite various attempts to improve inclusion rates, the necessary number of participants was not recruited. Seven hundred thirty-one participants were included: 325 in the depression trial and 406 in the anxiety trials. The Collabri-model was implemented, demonstrating good fidelity to core model elements. In favor of CC, we found a statistically significant difference between depression scores at 6-months’ follow-up in the depression trial. The difference was not significant at 15-months’ follow-up. The anxiety trials were pooled for data analysis due to inadequate sample sizes. At 6- and 15-months’ follow-up, there was a difference in anxiety symptoms favoring CC. These differences were not statistically significant. The ICER was 58,280 Euro per QALY.

**Conclusions:**

At 6 months, a significant difference between groups was found in the depression trial, but not in the pooled anxiety trial. However, these results should be cautiously interpreted as there is a risk of selection bias and lacking statistical power.

**Trial registration:**

ClinicalTrials.gov, ID: NCT02678624 and NCT02678845. Retrospectively registered on 7 February 2016.

**Supplementary Information:**

The online version contains supplementary material available at 10.1186/s12875-020-01299-3.

## Background

Depression and anxiety are common and disabling disorders [1], and most people diagnosed with depression and anxiety are treated in primary care [2]. Research suggests that collaborative care can be a useful organizational model for treating depression and anxiety disorders in this setting [3–5]. In collaborative care interventions, a primary care provider and one or more professionals are involved in providing care and proactive follow-up based on structured and evidence-based care plans [3]. At the same time, mechanisms to enhance communication between providers are introduced [3]. A meta-analysis from 2012 found that collaborative care was associated with larger short-, medium- and long-term improvements in symptoms compared with usual care for people with depression and anxiety [3]. However, most trials were conducted in the United States, and few included participants with anxiety disorders. The authors emphasized a need for more research in collaborative care for anxiety disorders, and that the findings should be interpreted more cautiously in settings different from that of the United States [3]. A subsequent systematic review and meta-analysis, including depression trials in European countries, showed that collaborative care also seems to be more effective than usual care in improving depression scores outside the United States [4]. In 2016, a meta-analysis focusing solely on collaborative care for anxiety disorders also found that collaborative care showed greater effects than usual care [5]. Until recently, no collaborative care trials have been conducted in Scandinavia. However, in 2018, a Swedish cluster-randomized collaborative care trial for depression showed a reduction in depression scores at 3- and 6-months’ follow-up, which was significantly greater in the intervention group vs. the control group when measured by MADRS-S but not by BDI-II [6].

In order to evaluate the effects of collaborative care in a Danish setting, the Collabri-model for collaborative care was developed in 2014 and subsequently tested. In this paper, we present results from 6- and 15-months’ follow-up of four cluster-randomized trials aiming at people with depression, panic disorder, generalized anxiety disorder, and social anxiety disorder in general practice. The hypothesis was that collaborative care would be superior to treatment-as-usual in reducing symptoms of depression in the depression trial and reducing anxiety symptoms in the anxiety trials. Ultimately, the trials failed because of failure to include participants and potential selection bias, despite randomization. Thus, we also provide insights into the lessons learned while conducting these trials.

## Methods

### Design

The Collabri trials were designed as four cluster-randomized, researcher-blinded, superiority trials evaluating the effects of collaborative care according to the Collabri-model compared to treatment-as-usual for patients with depression, generalized anxiety disorder, panic disorder, and social anxiety disorder. The design is described in more detail in two study design publications [7, 8]. The study adheres to CONSORT guidelines, and the Regional Ethics Committees in the Capital Region of Denmark approved the trial protocol.

### Recruitment of general practitioners and randomization

The random cluster allocation sequence was externally computer-generated by The Research Centre for Prevention and Health in the Capital Region of Denmark. One cluster consisted of a provider number in general practice, corresponding to one or more general practitioners (GPs). Patients were allocated after cluster-randomization to the same group as their GP/GPs. Cluster-randomization was chosen to avoid the risk of contamination bias. GPs in the Capital Region of Denmark (except the island of Bornholm) were invited to join the study through letters.

A total of 53 clusters were randomized during three rounds using simple randomization and an allocation ratio of 1:1 in the two first rounds, and an allocation ratio of 3:1 (control:collaborative care) in the third, including four clusters. The randomization was stratified by two geographical areas in the first round and three in the second. Randomization details are updated from previous descriptions [8]. A sub-study (nested study) investigated two methods of depression detection within the depression trial. Hence, GPs were additionally randomized into one of these detection methods. Findings from this study will be presented elsewhere.

### Recruitment of patients

GPs recruited participants and referred them to the study. GPs were encouraged to identify participants with depression according to their detection allocation and to use assessment tools in line with guidelines [9] when identifying participants with anxiety. GPs provided written and verbal information to patients and obtained oral and written consent. The GPs’ referral diagnosis was validated by a research assistant at a telephone interview with the patient using the MINI International Neuropsychiatric Interview (MINI) for DSM IV [10] and ICD-10 specific questions. In-and exclusion criteria were assessed by the GP and/or research assistant, and those included were sent a baseline questionnaire. If written consent was not received before the telephone interview, this was subsequently obtained. In case of a discrepancy between referral diagnosis and the research assistant’s assessment, the GP and project psychiatrist reached an agreement based on a discussion.

### Population

Patients were included in one of the four trial populations if they were registered at a participating GP, met the *International Classification of Diseases 10th edition (ICD-10)* diagnostic criteria for depression (F32–33), generalized anxiety disorder (F41.1), panic disorder (F41.0) or social anxiety disorder (F40.1), were at least 18 years old, spoke Danish and provided written consent. Patients were excluded if they had a dementia diagnosis or an unstable medical condition. Further exclusion criteria were pregnancy, medical/psychological treatment for anxiety or depression within the past 6 months, a pending disability pension application, referral to secondary mental health care, bipolar disorder, current psychotic condition, obsessive-compulsive disorder, high suicide risk, post-traumatic stress disorder, or substance abuse that would hinder participation. Additionally, patients of GPs allocated to the collaborative care intervention were excluded if they preferred treatment through the publicly subsidized psychologist program rather than collaborative care.

### Blinding

While conducting eligibility interviews and during the data collection phase, researchers were blinded to the participants’ and GP’s allocation. Researchers were also supposed to be blinded in the analysis- and concluding phase. However, due to a heavily skewed distribution between allocation groups, it was not possible to maintain this blinding. Furthermore, intervention staff in the collaborative care group, patients and GPs could not be blinded to the intervention, which is a general challenge when investigating psychosocial interventions.

### Interventions

#### The Collabri-model of collaborative care

While building on recommendations from a systematic literature review [11], the Collabri intervention further met four criteria often used to define collaborative care [3], but originally proposed to describe complex system-level interventions [12]: a multi-professional approach to care; enhanced inter-professional communication; scheduled follow-ups; and a structured management plan. GPs collaborated with a team of mental health specialists, including two psychiatrists and eight care managers employed by Mental Health Services in the Capital Region of Denmark. The group of care managers had a bachelor-level health care education and included nurses and an occupational therapist. They all had experience from working in mental health services and had taken a one-year or equivalent education of cognitive behavioral therapy (CBT). Care managers, psychiatrists, and GPs in the collaborative care group were trained in the model principles. Psychiatrists provided planned and ad hoc supervision of care managers and GPs. CBT supervision of care managers was introduced twice a month after trial commencement, as care managers requested this.

In around half of the GP practices, care managers had access to a consultation room in the practice. If not, care managers and patients met at facilities in the municipality or at a mental health center. Care managers’ caseload was predicted to be around 25; however, this was rarely reached because of lacking referrals. Each care manager collaborated with 3–5 GPs to provide appropriate treatment and close follow-up to assess progress. Treatment modalities (psychoeducation, CBT, and medication) were suggested according to disease-specific stepped-care algorithms, where care managers provided psychoeducation and CBT. The GP had the overall treatment responsibility and prescribed medication if this was indicated. For different reasons, group-based psychoeducation was only available initially in the trial period, whereas one-on-one psychoeducation and psychoeducation as part of CBT were offered throughout the trial period. A fidelity scale was developed to ensure the internal validity of the Collabri-model, and evaluations were carried out twice during the intervention period.

#### Treatment-as-usual

GPs in the treatment-as-usual group managed the participants’ care as they usually did. Clinical guidelines from the Danish Health Authority and the Danish College of General Practitioners were available for guidance, including recommendations on detection, diagnosis, treatment, and referral to specialized care [9, 13–15]. Treatment could vary between GPs as the guidelines only provide recommendations. As an example, interventions could include GPs managing care by providing psychoeducation and support, talking therapy, medication, or a combination. GPs could refer patients to a psychiatrist or mental health services free of charge for the patient or a psychologist, partly publicly subsidized.

### Outcome measurements and other data

All self-reported outcomes were assessed at baseline and after 6 and 15 months. Interviewer-rated measures were obtained at the eligibility interview and after 6 and 15 months. Participants were assessed at baseline using the Standardised Assessment of Personality: Abbreviated Scale (SAPAS) [16], while other baseline demographic data were obtained from Statistics Denmark [17]. The primary outcome was depression symptoms (Beck Depression Inventory (BDI-II)) [18] at 6-months’ follow-up in the depression trial. The primary outcome in the anxiety trials was self-reported anxiety symptoms (Beck Anxiety Inventory (BAI)) [19] at 6-months’ follow-up. See Table 1 for an overview of secondary-, explorative-, and safety measures.

**Table 1:** Overview of data

	Data collection method	6-months’ follow-up		15-months’ follow-up	
		Depression trial	Anxiety trial	Depression trial	Anxiety trial
Primary outcomes					
BDI-II	Self-reported	x			
BAI	Self-reported		x		
Secondary outcomes					
BDI-II	Self-reported		x	x	
BAI	Self-reported	x			x
SCL-90-R^a^ [20]	Self-reported	x	x	x	x
GAF [21]	Research assistant	x	x	x	x
Explorative outcomes					
BDI-II	Self-reported				x
BAI	Self-reported			x	
The Diagnostic Apathia Scale [22]	Research assistant	x	x	x	x
PSP [23]	Research assistant	x	x	x	x
SDS [24]	Self-reported	x	x	x	x
WHO-5 [25]	Self-reported	x	x	x	x
Personal Control^b^ [26]	Self-reported	x	x	x	x
Control/Manage Depression^c^ [27]	Self-reported	x	x	x	x
Obtain Help from Community, Family, Friends^c^ [27]	Self-reported	x	x	x	x
EQ-5D-3 L [28]	Self-reported	x	x	x	x
PRISE^d^ [29]	Self-reported	x	x	x	x
CSQ-8 [30]	Self-reported	x	x		
INSPIRE-S [31]	Self-reported	x	x		
INSPIRE-R [31]	Self-reported	x	x		
Weeks on sick leave benefits	DREAM register [32]	x	x	x	x
Proportion receiving sick leave benefits	DREAM register [32]	x	x	x	x
Psychiatric outpatient contacts	National Patient Register [33]	x	x	x	x
Safety measures and medication use					
Deaths by suicide and other reasons	Danish Register of Cause of Death [34]	x	x	x	x
Life-threatening conditions^e^	Charlson Comorbidity Index [35]	x	x	x	x
Somatic outpatient visits	National Patient Register [33]	x	x	x	x
Somatic inpatient days and admissions	National Patient Register [33]	x	x	x	x
Psychiatric inpatient days/admissions	National Patient Register [33]	x	x	x	x
Use of medication	Danish Register for Prescription medicine [36]	x	x		

*Abbreviations*: *BDI-II* Beck Depression Inventory-II, *BAI* Beck Anxiety Inventory, *CSQ-8* Client Satisfaction Questionnaire, *GAF-F* Global Assessment of Functioning, *EQ-5D-3L* EuroQol Five Dimensions Questionnaire with Three Levels, *INSPIRE-S* Recovery support from staff (Support), *INSPIRE-R* Recovery support from staff (Relationship), *PRISE* Patient Rated Inventory of Side Effects, *PSP* Personal and Social Performance Scale, *SCL-90-R* Symptom Checklist-90-Revised, *SDS* Sheehan Disability Scale, *WHO-5* World Health Organization-5 Well-Being Index

^a^ SCL-90-R was modified slightly as a reference period of two weeks was used instead of one week. ^b^ *S*ubscale from the Illness Perception Questionnaire-Revised (IPQ-R). ^c^ Subscale from the Chronic Disease Self-Efficacy Scales. ^d^ Side effects were reported for the proportion of participants who used medication. ^e^ Life-threatening conditions defined by the Charlson Comorbidity Index were assessed using action diagnoses (ICD codes) from the National Patient Register [33]

Specifications and corrections should be made to the two study design papers [7, 8]. For explorative subgroup analyses, we intended to obtain information about somatic comorbidities from GPs. Due to inadequate data and lack of statistical power analyses were, however, not made. Additionally, planned subgroup analyses for personality disorder were not performed because of a lack of statistical power. Sick leave benefits were by mistake included as safety measures. Sick leave is reported only as an explorative outcome. No other social services than sick leave benefits are included as an explorative outcome. Besides being reported as a safety measure, the number of psychiatric outpatient services is also reported as an explorative outcome. As the risk of suicide was only examined for all participants at baseline, this was not applicable as a safety measure as otherwise described. Instead, we distinguish between deaths from suicide and other reasons, using deaths from suicide as a safety measure. Medication use for anxiety and depression was used to describe treatment during the intervention period and was not reported at 15 months.

### Sample size calculations

Sample size calculations for primary outcomes using the program *PS: Power and Sample Size Calculation* showed that 364 participants should be included in each of the three anxiety trials, and 328 should be included in the depression trial. The sample size was adjusted to 480 in the depression trial because an additional study described elsewhere [7] was nested in the trial. Calculations were based on: a clinically relevant difference between groups of 4 points on BDI-II and BAI [37–39]; a standard deviation of 11 for BDI-II [37, 38, 40–43] and 12 for BAI [42–44]; a probability of type I error of 0.05 and a power of 0.8. The formula: 1 + (cluster size – 1) x ICC was used to estimate the design effect. The cluster size was 8 in the anxiety trials and 10 in the depression trial. The ICC was set at 0.04 [45]. Cluster-corrected sample sizes were found by multiplying the design effect with the sample size found via PS.

### Statistical analyses

Outcomes were assessed as differences between groups at follow-up, were based on intention-to-treat analysis [46], and all analyses accounted for cluster-randomization. As planned, we used linear mixed models to compare questionnaire-based effects: cluster level and participants were considered as random effects while time was set as a fixed effect. Generalized linear models were not used as wrongly stated elsewhere [8]. The stratification variable of the geographical area was included in all analyses. Methods for analyzing register-based data were not prespecified. However, continuous data were analyzed using Poisson regression, and logistic regression was used for dichotomous measures. As planned, 15-months’ follow-up data were analyzed using repeated measures with an unstructured covariance matrix. Because of missing data at all time points, questionnaire data were imputed (m = 100) using multivariate normal regression imputation (MCMC) under the assumption that data were “missing at random”.

Post hoc analyses were also performed. Due to smaller than expected sample sizes, we pooled results from the three anxiety populations to increase statistical power. We estimated statistical power and effect sizes (Cohen’s d) based on the primary outcomes at 6-months’ follow-up. In the depression trial, we used sample size simulation to estimate what the mean BDI-II score of the lacking participants in the control group should have been for the clinically and statistically significant differences to disappear. We assessed change from baseline to 6-months’ follow-up for all outcomes in the collaborative care groups. Finally, we estimated the proportion of participants in symptom remission, defined by a score of 13 or less on BDI-II in the depression trial and nine or less on BAI in the anxiety trial [18, 47].

### Health economic evaluation

The collaborative care intervention’s cost-effectiveness was assessed for a pooled group of participants with anxiety and depression, consisting of participants who had filled out the EQ-5D-3L questionnaire: 627 in the collaborative care group and 80 in the control group. We calculated costs from a public expense perspective with a time horizon of 6 months.

In both groups costs concerning healthcare usage and social benefits were calculated using the following information: hospital contacts and mental health outpatient services obtained from the National Patient Registry [33]; contacts with privately practicing health professionals in primary care obtained from the Danish National Health Service Register [48]; use of prescription drugs derived from the Danish National Prescription Registry [36]; and use of social benefits obtained from the DREAM database [32]. Collaborative care-related costs were estimated using data from the trials.

The cost development in the collaborative care group was calculated as the costs from baseline to 6-months’ follow-up minus the costs 6 months prior to inclusion. A similar measure was computed for the control group. The difference between the two differences was considered as the additional cost of the collaborative care intervention.

The health-related effects of the groups were measured in Quality Adjusted Life Years (QALYs). QALYs were based on the EQ-5D-3L questionnaire [28] completed by participants at baseline and 6-months’ follow-up. Means were calculated using the Danish preference weighting [49]. QALYs were estimated using complete case analysis adjusted for baseline differences. Robust T-test was used to assess differences between QALYs. Finally, the Incremental Cost-Effectiveness Ratio (ICER) was calculated as additional costs in the collaborative care group divided by the difference in QALY between groups.

## Results

### Characteristics of participating general practitioners

**Fig. 1:**
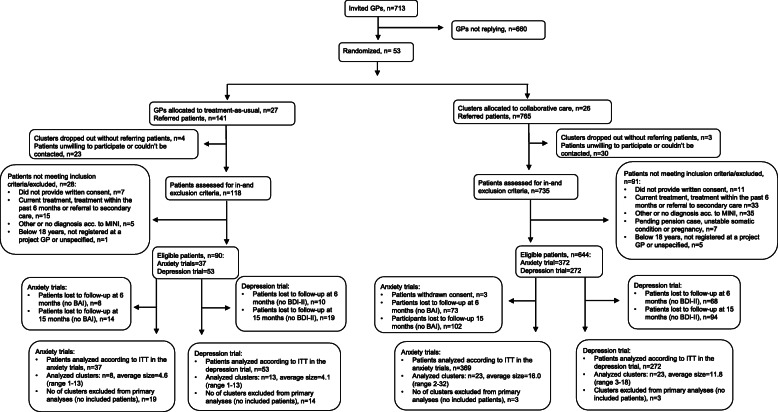
Flow chart

A total of 53 clusters (GPs) was recruited from May 2014 to July 2015. Most clusters were located in Copenhagen or surrounding areas and had one GP participating in the study. During the trial period, 7 clusters dropped out without having referred any participants. A total of 17 clusters did not refer patients included in the depression trial, and 22 clusters did not refer patients included in the anxiety trials (Fig. 1).

### Characteristics of participating patients

Recruitment of patients was started in November 2014 and ended in January 2017. In the depression trial, 325 participants were included; 272 in the collaborative care group and 53 in the control group. In the pooled anxiety trial, 406 participants were included; 369 in the collaborative care group and 37 in the control group (Fig. 1). Only around half of the expected total sample size was achieved, especially in the control group participants were lacking. Baseline assessment of the primary outcome was completed for 90% (*n* = 291) in the depression trial and 95% (*n* = 384) in the pooled anxiety trial. Most of the participants were women, and the mean age was 39 years and 36 years for participants with depression, respectively, anxiety disorders. Baseline characteristics are shown in Table 2.

**Table 2:** Baseline characteristics

	Depression trial		Pooled anxiety trial	
	CC (*n* = 272)	TAU (*n* = 53)	CC (*n* = 369)	TAU (*n* = 37)
Years of age, mean (SD)	39.0 (14.5)	39.1 (16.0)	36.3 (13.5)	36.1 (14.9)
Female, n (%)	177 (65.1)	40 (75.5)	239 (64.8)	31 (83.8)
Geographical area, n (%)				
Copenhagen area	126 (46.3)	25 (47.2)	183 (49.6)	21 (56.8)
Northern regional area	83 (30.5)	13 (24.5)	75 (20.3)	4 (10.8)
Western regional area	63 (23.2)	15 (28.3)	111 (30.1)	12 (32.4)
Marital status, n (%)^a^				
Not married	162 (59.6)	36 (67.9)	253 (68.6)	24 (64.9)
Married	77 (28.3)	13 (24.5)	97 (26.3)	10 (27.0)
Employment, n (%)^a^				
Employed	78 (28.7)	15 (28.3)	134 (36.3)	16 (43.2)
Studying	47 (17.3)	11 (20.8)	78 (21.1)	8 (21.6)
Unemployed	51 (18.8)	7 (13.2)	73 (19.8)	5 (13.5)
Receiving disability pension	4 (1.5)	<4^b^	7 (1.9)	<4^b^
Receiving retirement/early retirement pension	16 (5.9)	5 (9.4)	19 (5.1)	<4^b^
Receiving sickness benefits	73 (26.8)	13 (24.5)	54 (14.6)	5 (13.5)
Educational level, n (%)^a^				
Lower secondary education	63 (23.2)	11 (20.7)	79 (21.4)	5 (13.5)
Upper secondary education	108 (39.7)	25 (47.2)	151 (40.9)	19 (51.4)
Practical/occupationally specific, Bachelor or equivalent	53 (19.5)	11 (20.7)	85 (23.0)	6 (16.2)
Master, Doctoral or equivalent	11 (4.0)	<4^b^	33 (8.9)	4 (10.8)
Former psychiatric treatment, n (%)				
Outpatient MH treatment	39 (14.3%)	7 (13.2%)	51 (13.8%)	7 (18.9%)
Inpatient MH treatment	13 (4.8)	<4^b^	16 (4.3)	<4^b^
Primary diagnosis, n (%)				
Mild depression	37 (13.6)	9 (16.9)	–	–
Moderate depression	107 (39.3)	19 (35.8)	–	–
Severe depression	128 (47.1)	25 (47.2)	–	–
Generalized anxiety disorder	–	–	174 (47.2)	19 (51.4)
Panic disorder	–	–	124 (33.6)	12 (32.4)
Social anxiety disorder	–	–	71 (19.2)	6 (16.2)
Positive assessment of personality disorder (SAPAS > 2), n (%)	116 (43.9)	29 (58.0)	166 (46.8)	23 (65.7)

*Abbreviations*: *CC* Collaborative care, *SD* Standard deviation, *MH* Mental health, *SAPAS* Standardized Assessment of Personality, Abbreviated Scale, *TAU* Treatment-as-usual

Note: There were statistically significant baseline differences in the pooled anxiety trial for gender (*p* = 0.019) and assessment of personality disorder (*p* = 0.032)

^a^ Data was not available for all participants. Percentages are based on the total population. Thus, percentages do not add up to 100%. ^b^ Due to discretion, the exact number is not shown

### Treatment in the collaborative care group

Participants in the collaborative care group met with their care manager, on average, 8.7 times during an average period of 4.4 months. Most received psychoeducation alone or as part of CBT (Table 3). Respectively, 68 and 86% in the depression- and pooled anxiety trial received CBT as initial treatment. Around a third intensified treatment (stepped up) in the depression trial, equivalent to around a fourth in the pooled anxiety trial. Respectively, 21 and 16% in the depression and pooled anxiety trial were referred to specialist care. According to fidelity reports, the Collabri-model showed good implementation capability (Additional file 1).

**Table 3:** Treatment provided in the collaborative care group

	Depression trial (*n* = 271)	Pooled anxiety trial (*n* = 368)
Meetings between care manager and patient		
≥ One session (%)	262 (97)	361 (98)
No. of sessions, mean (min-max)	8.8 (0–24)	8.6 (0–19)
Length of treatment, days (months)	131 (4.4)	130 (4.3)
Initial treatment modalities, n (%)		
Psychoeducation	20 (7.4)	14 (3.8)
CBT	183 (67.5)	316 (85.9)
CBT and medication	50 (18.5)	16 (4.3)
Psychoeducation and medication	5 (1.8)	0 (0.0)
Other^a^	13 (4.8)	22 (6.0)
Total	271 (100.0)	368 (100.0)
Step ups, n (%)		
1 step up	84 (31.0)	65 (17.7)
> 1 step up	15 (5.5)	23 (6.3)
Treatment course, n (%)		
Completed treatment	166 (61.3)	236 (64.2)
Referred to specialist care before/during treatment	57 (21.0)	60 (16.3)
Outpatient psychiatric treatment	29 (50.9)	24 (40.0)
Municipal services/other	9 (15.8)	14 (23.3)
Private practicing psychologist	11 (19.3)	15 (25.0)
Private practicing psychiatrist	8 (14.0)	7 (11.7)
Drop out^b^	25 (9.2)	38 (10.3)
Other^c^	23 (8.5)	34 (9.2)
Total	271 (100.0)	368 (100.0)

*Abbreviations*: *CBT* Cognitive behavioral therapy

Note: Medication given on other indications than anxiety/depression and medication stated to be taken for less than three weeks (very few cases) was not included. Since there were missing data from one participant from each trial, data from these are not included in this table

^a^ Includes drop-out/referral before choosing treatment modality, monitoring/support, or no treatment information. ^b^ Primarily includes participants where contact could not be established before/during treatment, or where the participant explicitly stated that she/he did not want to start/continue treatment. ^c^ Primarily includes participants who changed address/GP or were referred back to GP without further information

### The depression trial

In the depression trial mean BDI-II scores decreased at 6-months’ follow-up from 28.4 (95% CI 27.2–29.6) at baseline to 13.3 (95% CI 12.0–14.6) in the collaborative care group and from 27.8 (95% CI 25.0–30.6) at baseline to 19.2 (95% CI 15.6–22.7) in the control group (Tables 4 and 5). The 6-months difference of − 5.9 points was statistically significant (*p* = 0.002), leading to an effect size of 0.52. The power was 66%. At 15-months’ follow-up, BDI-II scores decreased further to 11.8 (95% CI 10.5–13.1) in the collaborative care group and 14.7 (95% CI 11.0–18.4) in the control group. The 15-months difference of − 2.9 points was not statistically significant (*p* = 0.138).

**Table 4:** Questionnaire-based outcome means at baseline

	Depression trial		Pooled anxiety trial	
	CC (*n* = 272)	TAU (*n* = 53)	CC (*n* = 369)	TAU (*n* = 37)
BDI-II	28.4 (27.2–29.6)	27.8 (25.0–30.6)	18.1 (17.0–19.3)	18.1 (15.0–21.1)
BAI	17.5 (16.3–18.7)	17.9 (15.1–20.7)	21.8 (20.8–22.8)	21.2 (17.9–24.6)
SCL-90-R^a^	109.4 (103.2–115.7)	102.6 (87.2–117.9)	87.8 (81.9–93.6)	83.1 (62.9–103.2)
GAF	53.3 (51.9–54.8)	52.5 (49.5–55.5)	60.4 (59.3–61.6)	60.1 (57.0–63.2)
The Diagnostic Apathia Scale	8.4 (8.1–8.8)	8.8 (8.1–9.5)	5.9 (5.6–6.3)	5.9 (4.9–7.0)
PSP	55.9 (54.6–57.3)	53.8 (50.8–56.8)	62.3 (61.1–63.4)	62.0 (59.0–65.0)
SDS	18.3 (17.5–19.1)	16.3 (14.3–18.2)	13.5 (12.6–14.3)	14.3 (11.4–17.2)
WHO-5	22.2 (20.3–24.1)	24.0 (19.6–28.4)	34.9 (32.7–37.1)	35.9 (27.7–44.1)
Personal Control^b^	20.3 (19.8–20.8)	20.0 (19.1–20.8)	20.6 (20.2–21.0)	21.8 (20.5–23.1)
Control/Manage Depression^c^	4.4 (4.2–4.6)	4.7 (4.2–5.3)	5.6 (5.4–5.8)	5.4 (4.7–6.1)
Obtain Help from Community, Family, Friends^c^	5.8 (5.5–6.0)	5.4 (4.9–6.0)	6.5 (6.3–6.7)	6.8 (6.1–7.6)
EQ-5D-3 L	0.6 (0.6–0.7)	0.6 (0.6–0.7)	0.7 (0.7–0.7)	0.7 (0.6–0.7)
PRISE^d^	20.6 (19.1–22.1)	18.3 (14.9–21.8)	21.3 (19.4–23.2)	20.7 (15.0–26.3)

*Abbreviations*: *BDI-II* Beck Depression Inventory-II, *BAI* Beck Anxiety Inventory, *CC* Collaborative care, *CSQ-8* Client Satisfaction Questionnaire, *GAF-F* Global Assessment of Functioning, *EQ-5D-3L* EuroQol Five Dimensions Questionnaire with Three Levels, *PSP* Personal and Social Performance Scale, *PRISE* Patient Rated Inventory of Side Effects, *SCL-90-R* Symptom Checklist-90-Revised, *SDS* Sheehan Disability Scale, *TAU* Treatment-as-usual, *WHO-5* World Health Organization-5 Well-Being Index

Note: Means are based on observed cases and can vary from the total numbers indicated in the column headings. No statistically significant differences between groups were found, except for SDS in the depression trial (*p* = 0.046). In BDI-II, BAI, SCL-90-R, SDS, The Diagnostic Apathia Scale, and PRISE, lower scores are associated with a better outcome. In GAF, PSP, WHO-5, Personal control subscale from IPQ-R, Control/manage Depression subscale, Obtain Help from Community, Family, Friends subscale, and EQ-5D-3L higher scores are associated with a better outcome

^a^ SCL-90-R was modified slightly as a reference period of two weeks was used instead of one week. ^b^ *S*ubscale from the Illness Perception Questionnaire-Revised (IPQ-R). ^c^ Subscale from the Chronic Disease Self-Efficacy Scales. ^d^ Side effects were reported for the proportion of participants who used medication

**Table 5:** Questionnaire-based outcomes in the depression trial

	6-months’ follow-up				15-months’ follow-up		
	CC (*n* = 272)	TAU (*n* = 53)	*P*	Effect size	CC (*n* = 272)	TAU (*n* = 53)	*P*
Primary outcome							
BDI-II	13.3 (12.0–14.6)	19.2 (15.6–22.7)	0.002	0.52	11.8 (10.5–13.1)	14.7 (11.0–18.4)	0.138
Secondary outcomes							
BAI	11.0 (10.1–11.9)	14.5 (11.7–17.3)	0.024		9.3 (8.3–10.2)	11.6 (8.7–14.4)	0.132
SCL-90-R^a^	59.2 (53.5–65.0)	84.6 (63.4–105.8)	0.024		36.1 (30.1–42.2)	40.6 (16.8–64.4)	0.719
GAF	68.0 (66.3–69.6)	65.2 (61.1–69.3)	0.228		71.2 (69.3–73.0)	66.3 (60.1–72.6)	0.149
Explorative outcomes							
The Diagnostic Apathia Scale	3.4 (3.0–3.8)	4.3 (3.5–5.2)	0.062		3.1 (2.6–3.5)	3.9 (3.0–4.9)	0.095
PSP	68.7 (67.2–70.1)	66.9 (62.9–70.8)	0.397		71.3 (69.7–72.9)	68.7 (63.2–74.2)	0.383
SDS	9.5 (8.5–10.6)	11.0 (8.5–13.6)	0.291		6.7 (5.5–7.8)	9.1 (5.0–13.2)	0.266
WHO-5	54.2 (50.7–57.8)	46.7 (40.2–53.2)	0.044		54.3 (50.7–58.0)	52.6 (43.5–61.8)	0.738
Personal Control^b^	22.5 (21.9–23.2)	20.5 (18.9–22.1)	0.024		22.4 (21.8–23.1)	21.3 (19.9–22.7)	0.141
Control/Manage Depression^c^	6.3 (6.0–6.6)	5.3 (4.5–6.2)	0.033		6.2 (5.8–6.6)	5.9 (4.9–6.8)	0.467
Obtain Help from Community, Family, Friends^c^	6.5 (6.2–6.8)	5.2 (4.3–6.1)	0.010		6.3 (6.0–6.7)	6.1 (5.3–7.0)	0.697
EQ-5D-3 L	0.8 (0.8–0.8)	0.7 (0.6–0.8)	0.007		0.8 (0.8–0.9)	0.8 (0.7–0.8)	0.174
PRISE^d^	14.0 (12.2–15.9)	17.4 (13.5–21.3)	0.132		12.4 (10.5–14.4)	16.0 (11.1–20.9)	0.178
Remission, %	53.9 (47.1–60.7)	39.3 (25.2 53.3)	0.069		61.9 (55.2–68.5)	53.5 (37.9–69.1)	0.310
INSPIRE-S	74.1 (70.1–78.1)	50.3 (37.9–62.7)	< 0.001		–	–	
INSPIRE-R	84.7 (81.9–87.6)	60.8 (50.9–70.6)	< 0.001		–	–	
CSQ-8	26.7 (26.0–27.3)	21.6 (19.8–23.3)	< 0.001		–	–	

*Abbreviations*: *BDI-II* Beck Depression Inventory-II, *BAI* Beck Anxiety Inventory, *CC* Collaborative care, *CSQ-8* Client Satisfaction Questionnaire, *GAF-F* Global Assessment of Functioning, *EQ-5D-3L* EuroQol Five Dimensions Questionnaire with Three Levels, *INSPIRE-S* Recovery support from staff (Support), *INSPIRE-R* Recovery support from staff (Relationship), *PRISE* Patient Rated Inventory of Side Effects, *PSP* Personal and Social Performance Scale, *SCL-90-R* Symptom Checklist-90-Revised, *SDS* Sheehan Disability Scale, *TAU* Treatment-as-usual, *WHO-5* World Health Organization-5 Well-Being Index

Note: Means are estimated based on imputed data. In BDI-II, BAI, SCL-90-R, SDS, The Diagnostic Apathia Scale, and PRISE, lower scores are associated with a better outcome. In GAF, PSP, WHO-5, Personal control subscale from IPQ-R, Control/manage Depression subscale, Obtain Help from Community, Family, Friends subscale, EQ-5D-3L, CSQ-8, INSPIRE-S, and INSPIRE-R higher scores are associated with a better outcome

^a^ SCL-90-R was modified slightly as a reference period of two weeks was used instead of one week. ^b^ *S*ubscale from the Illness Perception Questionnaire-Revised (IPQ-R). ^c^ Subscale from the Chronic Disease Self-Efficacy Scales. ^d^ Side effects were reported for the proportion of participants who used medication

There were statistically significant differences between groups at 6-months’ follow-up on several self-reported outcomes favoring collaborative care (Table 5). In Tables 6 and 7, results from register-based explorative analyses are displayed. From baseline to 6-months’ follow-up, the collaborative care group showed statistically significant improvements on all self-reported outcomes (Additional file 2). Post hoc analyses showed that the 111 participants in the control group, who were missing to achieve the planned number of participants in the control group, should have had a mean BDI-II score below 16.4 at 6-months’ follow-up to nullify the clinically relevant difference of 4 points. The statistically significant difference would disappear if the missing participants had a BDI-II mean score below 14.9.

**Table 6:** Explorative outcomes and measures of harms in the depression and pooled anxiety trial

		6-months’ follow-up			15-months’ follow-up		
		IR	IRR (95% CI)	*P*	IR	IRR (95% CI)	*P*
Depression trial							
Weeks on sick leave benefit	TAU	6.1 (3.7–8.5)	1		8.8 (4.8–12.7)	1	
	CC	6.1 (5.0–7.1)	1.0 (0.7–1.5)	0.990	9.4 (7.8–11.1)	1.1 (0.7–1.7)	0.756
Psychiatric outpatient visits	TAU	0.5 (0.0–1.1)	1		1.4 (0.0–3.0)	1	
	CC	0.3 (0.2–0.4)	0.5 (0.2–1.7)	0.277	1.8 (1.1–2.5)	1.3 (0.4–4.1)	0.677
Psychiatric inpatient days	TAU	0.7 (0.0–1.5)	1		0.7 (0.0–1.8)	1	
	CC	0.1 (0.0–0.1)	0.1 (0.01–0.7)	0.019	0.2 (0.0–0.6)	0.4 (0.0–3.4)	0.373
Psychiatric admissions	TAU	0.02 (0.0–0.1)	1		0.04 (0.0–0.1)	1	
	CC	0.01 (0.0–0.03)	0.8 (0.1–5.5)	0.781	0.03 (0.0–0.1)	0.6 (0.1–4.2)	0.645
Somatic outpatient visits	TAU	0.4 (0.1–0.8)	1		1.7 (0.5–2.9)	1	
	CC	0.8 (0.6–1.1)	1.8 (0.7–4.4)	0.198	2.1 (1.5–2.6)	1.2 (0.6–2.5)	0.621
Pooled anxiety trial							
Weeks on sick leave benefit	TAU	1.4 (0.0–3.2)	1		2.8 (0.0–5.8)	1	
	CC	3.4 (2.7–4.1)	2.4 (0.7–8.7)	0.189	5.6 (4.2–7.0)	2.0 (0.6–6.4)	0.236
Psychiatric outpatient visits	TAU	0.4 (0.0–0.8)	1		1.1 (0.0–2.3)	1	
	CC	0.2 (0.1–0.3)	0.5 (0.1–1.5)	0.201	1.4 (0.8–2.0)	1.3 (0.4–4.4)	0.663
Psychiatric inpatient days	TAU	0.04 (0.0–0.1)	1		0.04 (0.0–0.2)	1	
	CC	0.1 (0.0–0.3)	2.6 (0.2–43.0)	0.512	0.11 (0.0–0.3)	2.7 (0.2–44.3)	0.481
Psychiatric admissions	TAU	0.03 (0.0–0.1)	1		0.04 (0.0–0.1)	1	
	CC	0.02 (0.0–0.03)	0.6 (0.1–5.0)	0.608	0.03 (0.0–0.05)	0.7 (0.1–7.0)	0.785
Somatic outpatient visits	TAU	0.3 (0.1–0.6)	1		1.1 (0.7–1.6)	1	
	CC	0.6 (0.5–0.7)	1.7 (0.7–4.1)	0.213	1.8 (1.5–2.1)	1.6 (1.0–2.5)	0.052

*Abbreviations*: *CC* Collaborative care, *TAU* Treatment-as-usual, *IR* Incidence rate, *IRR* Incidence rate ratio

**Table 7:** Deaths, use of medication for anxiety/depression and sick leave benefits

	6-months’ follow-up			15-months’ follow-up		
Depression trial, n (%)	CC (*n* = 272)	TAU (*n* = 53)	*P*	CC (*n* = 272)	TAU (*n* = 53)	*P*
Deaths, suicide	0	0		0	0	
Deaths, other reasons	0	0		0	0	
Use of medication	126 (46.3)	30 (56.7)	0.218			
Proportion receiving sick leave benefits	35 (12.9)	7 (13.2)	0.945	18 (6.6)	<4^a^	0.770
Pooled anxiety trial, n (%)	CC (*n* = 369)	TAU (*n* = 37)	*P*	CC (*n* = 369)	TAU (*n* = 37)	*P*
Deaths, suicide	0	<4^a^		0	0	
Deaths, other reasons	0	0		0	0	
Use of medication	96 (26.0)	14 (37.8)	0.008			
Proportion receiving sick leave benefits	30 (8.1)	<4^a^	0.258	13 (3.5)	<4^a^	0.751

*Abbreviations*. *CC* Collaborative care, *TAU* Treatment-as-usual

^a^ Due to discretion the exact number is not shown

### The pooled anxiety trial

In the pooled anxiety trial, the mean BAI scores decreased from 21.8 (95% CI 20.8–22.8) to 11.5 (95% CI 10.6–12.4) at 6 months in the collaborative care group and from 21.2 (95% CI 17.9–24.6) to 14.6 (95% CI 9.9–19.3) in the control group. The − 3.1 points difference at 6-months’ follow-up was not statistically significant (*p* = 0.206) (Tables 4 and 8). This difference was equivalent to an effect size of 0.33. The statistical power was 72%. At 15-months’ follow-up, the mean BAI score was 11.0 (95% CI 10.2–11.9) in the collaborative care group and 12.7 (95% CI 10.2–15.3) in the control group. This difference was not statistically significant (*p* = 0.209).

**Table 8:** Questionnaire-based outcomes in the pooled anxiety trial

	6-months’ follow-up				15-months’ follow-up		
	CC (*n* = 369)	TAU (*n* = 37)	*P*	Effect size	CC (*n* = 369)	TAU (*n* = 37)	*P*
Primary outcome							
BAI	11.5 (10.6–12.4)	14.6 (9.9–19.3)	0.206	0.33	11.0 (10.2–11.9)	12.7 (10.2–15.3)	0.209
Secondary outcomes							
BDI-II	9.2 (8.3–10.1)	11.3 (6.3–16.3)	0.418		9.1 (8.3–9.9)	10.9 (7.7–14.0)	0.295
SCL-90-R^a^	48.6 (43.8–53.3)	64.9 (40.5–89.4)	0.200		47.4 (42.6–52.0)	42.7 (23.1–62.2)	0.657
GAF	72.5 (70.9–74.1)	72.9 (68.8–77.0)	0.855		74.8 (73.3–76.3)	80.3 (71.6–89.0)	0.224
Explorative outcomes							
The Diagnostic Apathia Scale	2.5 (2.2–2.8)	2.4 (1.3–3.5)	0.843		2.1 (1.9–2.4)	2.1 (1.0–3.3)	0.961
PSP	72.6 (71.2–74.1)	72.7 (69.3–76.2)	0.951		75.3 (73.7–76.9)	82.1 (65.5–98.8)	0.417
SDS	6.2 (5.4–7.0)	7.1 (4.6–9.7)	0.485		5.5 (4.6–6.3)	7.1 (4.5–9.7)	0.243
WHO-5	59.1 (56.7–61.4)	59.2 (48.9–69.3)	0.995		61.7 (59.0–64.5)	53.0 (37.8–68.2)	0.271
Personal Control^b^	23.3 (22.8–23.8)	22.2 (20.2–24.2)	0.282		19.9 (19.6–20.2)	20.3 (19.3–21.2)	0.465
Control/Manage Depression^c^	7.1 (6.9–7.3)	6.0 (4.9–7.0)	0.031		7.2 (6.9–7.4)	7.1 (6.3–7.9)	0.885
Obtain Help from Community, Family, Friends^c^	7.2 (6.9–7.4)	6.9 (5.8–8.0)	0.637		7.4 (7.2–7.6)	7.3 (6.6–8.1)	0.848
EQ-5D-3 L	0.84 (0.82–0.86)	0.78 (0.67–0.90)	0.294		0.8 (0.8–0.9)	0.9 (0.8–0.9)	0.536
PRISE^d^	15.0 (13.0–16.9)	14.0 (5.3–22.7)	0.828		12.1 (9.9–14.3)	15.4 (0.8–30.6)	0.662
Remission, %	45.7 (40.1–51.2)	39.2 (22.3–56.2)	0.481		49.0 (43.2–54.9)	42.3 (24.6–60.0)	0.442
INSPIRE-S	74.0 (70.7–77.3)	67.7 (50.0–85.4)	0.488		–	–	
INSPIRE-R	85.8 (83.6–88.0)	82.4 (69.7–95.1)	0.604		–	–	
CSQ-8	26.7 (26.2–27.4)	23.9 (17.5–30.2)	0.368		–	–	

*Abbreviations*: *BDI-II* Beck Depression Inventory II, *BAI* Beck Anxiety Inventory, *CC* Collaborative care, *CSQ-8* Client Satisfaction Questionnaire, *EQ-5D-3L* EuroQol Five Dimensions Questionnaire with Three Levels, *GAF-F* Global Assessment of Functioning, *INSPIRE-S* Recovery support from staff (Support), *INSPIRE-R* Recovery support from staff (Relationship), *PRISE* Patient Rated Inventory of Side Effects, *PSP* Personal and Social Performance Scale, *SCL-90-R* Symptom Checklist-90-Revised, *SDS* Sheehan Disability Scale, *TAU* Treatment-as-usual, *WHO-5* World Health Organization-5 Well-Being Index

Note: Means are estimated based on imputed data. In BDI-II, BAI, SCL-90-R, SDS, The Diagnostic Apathia Scale, and PRISE, lower scores are associated with a better outcome. In GAF, PSP, WHO-5, Personal control subscale from IPQ-R, Control/manage Depression subscale. Obtain Help from Community, Family, Friends subscale, EQ-5D-3L, CSQ-8, INSPIRE-S, and INSPIRE-R higher scores are associated with a better outcome

^a^ SCL-90-R was modified slightly as a reference period of two weeks was used instead of one week. ^b^ *S*ubscale from the Illness Perception Questionnaire-Revised (IPQ-R). ^c^ Subscale from the Chronic Disease Self-Efficacy Scales. ^d^ Side effects were reported for the proportion of participants who used medication

In Tables 6 and 7, results from register-based explorative analyses are displayed. From baseline to 6-months’ follow-up, the collaborative care group showed statistically significant improvements in all outcomes (Additional file 2). Results for each of the three anxiety trials are included in Additional files 3 and 4.

### Harms

There were no statistically significant differences indicating that the collaborative care group had more deaths, psychiatric bed-days, or -admissions, or more somatic outpatient visits than the treatment-as-usual-group (Tables 6 and 7). It was not possible to conduct analyses regarding life-threatening conditions, somatic admissions, and somatic bed-days because of too few cases.

### Health economic evaluation

The additional costs of the collaborative care intervention were estimated to be 1457 Euro (Additional file 5). This difference in costs was not statistically significant. Analyses showed a statistically significant difference between groups of 0.025 QALY (*p* = 0.006) in favor of the collaborative care group (Additional file 6). The ICER was estimated to be 58,280 Euro per QALY, suggesting that collaborative care is not cost-effective within a 6 months’ timeframe.

## Discussion

In the present depression study, the estimated effect size of 0.52 based on BDI-II is comparable to or even higher than standardized mean differences (SMDs) found in meta-analyses ranging from 0.19 (0–3-months’ follow-up) [4] to 0.34 (0–6-months’ follow-up) [3]. The effect size of 0.33 based on BAI in the pooled anxiety trial is also comparable to SMDs found in meta-analyses of 0.30 (0–6-months’ follow-up) [3] and 0.35 (0–12-months’ follow-up) [5]. However, because of the high risk of selection bias in our study, effect sizes could be inflated.

### Lessons learned

While monitoring recruitment data, we found different referral patterns across GPs. Some GPs, regardless of allocation, referred numbers close to what was expected, some referred less, and in the control group, several GPs never got started referring. Generally, GPs referred patients with anxiety to a lesser extent than predicted. Consequently, even though baseline data did not reveal major differences between groups, we assume that there is a risk of selection bias. Baseline validation of participants’ diagnoses helped ensure eligibility regardless of allocation; however, we did not have any procedure to ensure that all eligible individuals were asked to participate. Different aspects of the design and its underlying assumptions could have influenced the referral pattern: a recruitment strategy resting solely on GPs to invite patients to participate, GPs’ different perceptions of obstacles to refer, and lower than expected disease prevalence or disease detection in GPs’ practices.

We chose the strategy of GPs referring patients to the study as this was acceptable by GPs. However, other recruitment strategies were considered in the design phase, such as waiting room screening and implementation of pop-up windows in GPs’ medical records to remind them of potentially eligible participants. GPs objected to the method of waiting room screening, and even though we worked on making pop-up windows available, this strategy was at the last minute made impossible due to reasons unrelated to the project. While some cluster-randomized collaborative care studies have used a similar recruitment strategy to ours [50–52], other trials have recruited participants through annual health screenings or searches in medical records [53–55]. In our study, medical record screening would have required access to the GPs’ electronic record systems, which unfortunately was not an opportunity. Muntingh et al. used a combination of GP identification and medical record identification [56]. They found that participants in the collaborative care group were more often selected for the trial by their GP than in the control group, where a larger proportion was recruited from medical records. This, similarly to our study, suggests difficulties in recruiting participants from GPs allocated to a control group.

During the trial period, we attempted to improve intake rates by continuously encouraging GPs to refer to the project and prolonged the recruitment period. GPs received newsletters sharing updates and successes, posters were hung in GP’s waiting rooms, and project information was shared in newspapers. Primo 2016, we conducted an informal telephone survey with a sample of GPs across intervention groups to assess perceived obstacles for referring patients. Difficulties remembering to refer, concerns that it would be stressful for patients to participate (e.g., in the eligibility interview), issues related to the referral process, and presence of specific exclusion criteria were some of the obstacles mentioned. Similarly, other literature has found time constraints and clinicians’ concern for their patients as barriers for recruitment [57]. Also, narrow inclusion criteria have been reported to be associated with poor recruitment [58]. While we sought to respond to GPs’ needs and attempted to solve any uncertainties affecting their referral pattern, queries about removing exclusion criteria were not accommodated as the associated methodological disadvantages were considered larger than the anticipated benefits.

### Strengths and limitations

While many trials build their intervention on collaborative care principles, models can differ in additional content. Strengths of this study are that we provide an elaborate description of the collaborative care model components [7, 8] to ensure transparency, and we report on harms. Further, we developed a collaborative care model including elements shown to be associated with improved outcomes in previous studies [59, 60]. These are elements such as specialist supervision of care managers, recruiting care managers with experience from working in mental health services, and integrating the provision of a psychological intervention into the model [59, 60]. Twice during the project period, we monitored fidelity to the model to ensure that care managers, psychiatrists, and GPs delivered the intervention as intended. Other strengths were the externally conducted computer-based cluster-randomization, which ensured random and concealed allocation of GPs. The use of blinded assessment of the secondary outcome Global Assessment of Functioning and application of intention-to-treat analyses also decreased the risk of biased effect estimates.

Besides lacking statistical power and possible selection bias, there are other limitations to this study. It is a limitation that we have no information on the treatment given by providers such as private practicing psychologists or psychiatrists in the control group. From this, we could have assessed whether the type and amount of treatment differed between the collaborative care- and treatment-as-usual group. We were not able to blind participants, care managers, psychiatrists, or GPs to the allocation, and due to the skewed distribution between groups, we could not blind researchers when analyzing data or when writing the conclusion. Another limitation was that primary outcomes were self-reported and, therefore, not blinded, which could lead to overestimation of treatment effects. However, self-report measures mirror participants’ own perceptions of symptoms, which is also valuable seen from a recovery perspective. Although participants were recruited by GPs throughout the Capital Region of Denmark, there may be reduced external validity as GPs signed up voluntarily to participate. This could indicate an interest in common mental disorders or inter-sectoral collaboration, which may not be representative of the general GP population.

### Implications for research and practice

Feasibility- or pilot testing of the trial prior to commencement might have helped us identify recruitment problems at an earlier stage. A feasibility study aims to provide information about different trial processes [61]. A pilot study is frequently referred to as a small-scale version of the study one wishes to conduct and seek to test how the various processes work together [61]. Conducting pilot studies is no guarantee that recruitment will proceed successfully [62]. However, if we had completed pilot- or feasibility studies, some of the theory-based assumptions made while writing the protocol, such as prevalence estimations, could have been empirically validated.

There are examples of successfully completed cluster-randomized controlled trials [53, 63]; however, it is recognized that many cluster-randomized trials and RCTs, in general, have problems recruiting the predefined study sample [62, 64–66]. This is a problem of concern, as research questions consequently remain to be answered, or there can be delays in demonstrating important effects [67]. If data from unsuccessful trials are never published, this can further lead to publication bias within the specific research field. Because of the limitations of this study, there is still a need to examine the effects of collaborative care in a Danish setting. Therefore, two new trials, referred to as the Collabri Flex trials, have been initiated [68], and recruitment goals are reached. The Collabri Flex trials are based upon the knowledge gained from the Collabri Trials, and to achieve an equal distribution between groups, we randomized at the individual level. The effect results and a health economic evaluation of these trials will be reported elsewhere.

## Conclusion

Regrettably, due to limitations of the cluster-randomized design, we failed to carry out the effect trials as planned. For people with depression, we found a statistically significant difference between collaborative care and treatment-as-usual at 6-months’ follow-up in favor of collaborative care. For people with anxiety disorders, a non-significant difference between groups was found. Nevertheless, these results are limited by a lack of statistical power and possible selection bias. However, we succeeded in implementing the Collabri collaborative care model to provide patients with evidence-based treatment in line with guidelines in Danish general practices. Based on the results, we cannot rule out that collaborative care may be an effective way of organizing treatment in the Danish setting, but this hypothesis remains to be verified. Therefore, the Collabri trials act as the background for the Collabri Flex trials, and hence for improvement of future treatment of depression and anxiety disorders in primary care in Denmark.

## Supplementary Information

**Additional file 1: Table A1.** Themes and scores of fidelity reports.

**Additional file 2: Table A2.** Change in questionnaire-based outcomes in the CC groups from baseline to 6-months’ follow-up.

**Additional file 3: Table A3.** Questionnaire-based outcome means in the anxiety sub-trials at 6-months’ follow-up.

**Additional file 4: Table A4.** Questionnaire-based outcome means in the anxiety trials at 15-months’ follow-up.

**Additional file 5: Table A5.** Additional costs of collaborative care compared to treatment-as-usual.

**Additional file 6: Table A6.** Mean QALYs in the CC group and TAU group based on 6-months follow-up.

**Additional file 7.** Consort 2010 checklist of information to include when reporting a randomized trial.

## Data Availability

Due to the General Data Protection Regulation data is not available. Data can be retrieved upon reasonable request.

## Abbreviations

BDI-II: Beck Depression Inventory II
BAI: Beck Anxiety Inventory
CBT: Cognitive behavioral therapy
CC: Collaborative care
CSQ-8: Client Satisfaction Questionnaire
EQ-5D-3L: EuroQol Five Dimensions Questionnaire with Three Levels
GP: General practitioner
GAF-F: Global Assessment of Functioning
ICER: Incremental cost-effectiveness ratio
IPQ-R: Illness Perception Questionnaire-Revised
MINI: MINI International Neuropsychiatric Interview
PSP: Personal and Social Performance Scale
PRISE: Patient Rated Inventory of Side Effects
QALY: Quality adjusted life years
RCT: Randomized controlled trial
SD: Standard deviation
SAPAS: Standardized Assessment of Personality Abbreviated Scale
SDS: Sheehan Disability Scale
SCL-90-R: Symptom Checklist-90-Revised
SMD: Standardized mean difference
TAU: Treatment-as-usual
WHO-5: World Health Organization-5 Well-Being Index
