# Supplementary material for: Collaborative care for depression and anxiety disorders: results and lessons learned from the Danish cluster-randomized Collabri trials
Source: BMC Fam Pract. 2020 Nov 18;21:234. doi: 10.1186/s12875-020-01299-3 (PMC7673096; doi:10.1186/s12875-020-01299-3)
Supplement: Supplementary file 1 — Additional file 1: Table A1. Themes and scores of fidelity reports. [file 12875_2020_1299_MOESM1_ESM.docx]

Table A1. Themes and scores of fidelity reports

| Criteria | Fidelity 2015 | Fidelity 2016 |
| --- | --- | --- |
| Organization |  |  |
| Place for meetings between the care manager and patient: in general practice | 2 | 2 |
| The Collabri Team: minimum of two care managers and one part-time psychiatrist, supervision of care managers | 3 | 5 |
| Collaboration between GP, care manager, and psychiatrist | 5 | 4 |
| Meeting between the care manager and GP: weekly | 5 | 4 |
| Supervision by the GP: monthly by a psychiatrist | 2 | 4 |
| Collabri team management and anchoring: at a mental health center | 3 | 3 |
| Interdisciplinary record keeping: Collabri team access to medical record | 2 | 2 |
| Staff |  |  |
| Care manager caseload: maximum of 25 patients | 5 | 5 |
| Care manager’s professional skills: education, clinical experience | 5 | 5 |
| Care manager treatment generalist | 5 | 5 |
| Care Managers Working Time: 65% of the time is used on patient consultations | 3 | 2 |
| Clinical skills of supervising therapist: psychiatrist | 5 | 5 |
| CBT skills of supervising therapists | 3 | 4 |
| Psychiatrists working hours: minimum part-time employed | 5 | 5 |
| Treatment |  |  |
| Stepped care principle | 5 | 5 |
| Detection: GP uses a screening instrument for detection | 5 | 3 |
| Structured Treatment Guide – Medical treatment: algorithms are used | 5 | 5 |
| Structured Treatment Guide – Cognitive Behavioral Therapy: manuals are used | 5 | 4 |
| Structured Treatment Guide – Self-help Assistance: psychoeducation | 4 | 5 |
| Patient involvement: shared decision making | 5 | 4 |
| Involvement of relatives | 4 | 5 |
| Total fidelity score | 86 | 86 |

Abbreviations: CBT: Cognitive Behavioral Therapy, GP: General Practitioner.

Interpretation of total fidelity score: 97-105=Exemplary Collabri fidelity, 84-96=Good Collabri fidelity, 62-83=Fair Collabri fidelity, <61=Not Collabri fidelity
