# Supplementary material for: Collaborative care for depression and anxiety disorders: results and lessons learned from the Danish cluster-randomized Collabri trials
Source: BMC Fam Pract. 2020 Nov 18;21:234. doi: 10.1186/s12875-020-01299-3 (PMC7673096; doi:10.1186/s12875-020-01299-3)
Supplement: Supplementary file 2 — Additional file 2: Table A2. Change in questionnaire-based outcomes in the CC groups from baseline to 6-months’ follow-up. [file 12875_2020_1299_MOESM2_ESM.docx]

Table A2. Change in questionnaire-based outcomes in the CC groups from baseline to 6-months’ follow-up

|  | Depression trial | |  | Pooled anxiety trial | |  |  |
| --- | --- | --- | --- | --- | --- | --- | --- |
|  | Baseline | 6-months’ follow-up | P | Baseline | 6-months’ follow-up |  | P |
|  | Mean (95% CI) | Mean (95% CI) |  | Mean (95% CI) | Mean (95% CI) |  |  |
| Primary outcome |  |  |  |  |  |  |  |
| BDI-II | 28.3 (27.3-29.3) | 13.3 (11.9-14.6) | <0.001 |  |  |  |  |
| BAI |  |  |  | 21.8 (20.6-23.0) | 11.5 (10.6-12.4) |  | <0.001 |
| Secondary outcomes |  |  |  |  |  |  |  |
| BDI-II | - | - |  | 18.1 (16.8-19.4) | 9.2 (8.2-10.1) |  | <0.001 |
| BAI | 17.4 (16.3-18.5) | 11.0 (10.0-12.0) | <0.001 | - | - |  |  |
| SCL-90-R^a^ | 108.9 (102.9-114.9) | 59.2 (53.2-65.2) | <0.001 | 95.6 (89.0-102.2) | 48.4 (43.5-53.3) |  | <0.001 |
| GAF | 53.3 (51.8-54.9) | 68.0 (66.3-69.7) | <0.001 | 60.5 (59.0-61.9) | 72.6 (70.8-74.3) |  | <0.001 |
| Explorative outcomes |  |  |  |  |  |  |  |
| The Diagnostic Apathia Scale | 8.4 (8.1-8.8) | 3.4 (2.9-3.9) | <0.001 | 5.9 (5.4-6.4) | 2.5 (2.2-2.8) |  | <0.001 |
| PSP | 55.9 (54.4-57.5) | 68.7 (67.2-70.2) | <0.001 | 62.4 (61.1-63.7) | 72.7 (71.0-74.3) |  | <0.001 |
| SDS | 18.5 (17.7-19.3) | 9.5 (8.5-10.6) | <0.001 | 13.8 (13.0-14.6) | 6.2 (5.4-7.0) |  | <0.001 |
| WHO-5 | 22.1 (20.3-23.9) | 54.2 (50.6-57.8) | <0.001 | 35.1 (32.9-37.2) | 59.1 (56.7-61.4) |  | <0.001 |
| Personal Control^b^ | 20.3 (19.8-20.8) | 22.53 (21.9-23.2) | <0.001 | 20.6 (20.2-21.1) | 23.3 (22.7-23.9) |  | <0.001 |
| Control/Manage Depression^c^ | 4.4 (4.2-4.6) | 6.3 (6.0-6.6) | <0.001 | 5.6 (5.4-5.8) | 7.1 (6.9-7.3) |  | <0.001 |
| Obtain Help from Community, Family, Friends^c^ | 5.7 (5.5-6.0) | 6.5 (6.2-6.8) | <0.001 | 6.5 (6.3-6.8) | 7.2 (6.9-7.5) |  | <0.001 |
| EQ-5D-3 L | 0.62 (0.60-0.65) | 0.81 (0.79-0.84) | <0.001 | 0.70 (0.68-0.72) | 0.84 (0.82-0.86) |  | <0.001 |
| PRISE^d^ | 20.7 (19.3-22.2) | 13.9 (12.0-15.9) | <0.001 | 21.3 (19.6-22.9) | 15.0 (13.0-16.9) |  | <0.001 |

Abbreviations: BDI-II: Beck Depression Inventory-II, BAI: Beck Anxiety Inventory, CC: Collaborative care, CSQ-8: Client Satisfaction Questionnaire, EQ-5D-3L: EuroQol Five Dimensions Questionnaire with Three Levels, GAF-F: Global Assessment of Functioning, PRISE: Patient Rated Inventory of Side Effects, PSP: Personal and Social Performance Scale, SCL-90-R: Symptom Checklist-90-Revised, SDS: Sheehan Disability Scale, WHO-5: World Health Organization-5 Well-Being Index.

Note: In BDI-II, BAI, SCL-90-R, SDS, The Diagnostic Apathia Scale, and PRISE, lower scores are associated with a better outcome. In GAF, PSP, WHO-5, Personal control subscale from IPQ-R, Control/manage Depression subscale, Obtain Help from Community, Family, Friends subscale, and EQ-5D-3L, higher scores are associated with a better outcome.

^a^ SCL-90-R was modified slightly as a reference period of two weeks was used instead of one week. ^b^ Subscale from the Illness Perception Questionnaire-Revised (IPQ-R). ^c^ Subscale from the Chronic Disease Self-Efficacy Scales. ^d^ Side effects are reported for the proportion of participants who used medication.
