# Supplementary material for: Collaborative care for depression and anxiety disorders: results and lessons learned from the Danish cluster-randomized Collabri trials
Source: BMC Fam Pract. 2020 Nov 18;21:234. doi: 10.1186/s12875-020-01299-3 (PMC7673096; doi:10.1186/s12875-020-01299-3)
Supplement: Supplementary file 3 — Additional file 3: Table A3. Questionnaire-based outcome means in the anxiety sub-trials at 6-months’ follow-up. [file 12875_2020_1299_MOESM3_ESM.docx]

Table A3. Questionnaire-based outcome means in the anxiety trials at 6-months’ follow-up

|  | Generalized Anxiety Disorder | | | Panic Disorder | | | Social Anxiety Disorder | | |
| --- | --- | --- | --- | --- | --- | --- | --- | --- | --- |
|  | CC (n=174) | TAU (n=19) |  | CC (n=124) | TAU (n=12) |  | CC (n=71) | TAU (n=6) |  |
|  | Mean (95% CI) | Mean (95% CI) | P | Mean (95% CI) | Mean (95% CI) | P | Mean (95% CI) | Mean (95% CI) | P |
| Primary outcome | | | | | | | | | |
| BAI | 12.2 (11.1-13.3) | 14.7 (8.1-21.2) | 0.469 | 10.9 (9.6-12.2) | 12.9 (5.3-20.5) | 0.608 | 11.2 (9.1-13.4) | 15.4 (7.3-23.5) | 0.331 |
| Secondary outcomes | | | | | | | | | |
| BDI-II | 10.1 (8.8-11.3) | 11.5 (3.5-19.6) | 0.724 | 8.0 (6.5-9.5) | 9.6 (1.5-17.8) | 0.696 | 9.2 (7.5-11.0) | 12.8 (3.8-21-7) | 0.446 |
| SCL-90-R^a^ | 50.8 (45.3-56.3) | 61.1 (24.5-97.7) | 0.586 | 43.5 (36.5-50.5) | 64.9 (31.7-98.2) | 0.217 | 53.0 (41.9-64.0) | 65.7 (22.1-109.3) | 0.578 |
| GAF | 72.5 (70.3-74.6) | 71.9 (65.6-78.3) | 0.875 | 72.7 (70.3-75.0) | 75.8 (68.6-82.9) | 0.420 | 71.9 (67.7-76.2) | 71.6 (64.7-78.5) | 0.938 |
| Explorative outcomes | | | | | | | | | |
| The Diagnostic Apathia Scale | 2.9 (2.5-3.3) | 2.8 (1.4-4.2) | 0.939 | 2.2 (1.7-2.71) | 1.4 (0.0-3.0) | 0.354 | 2.3 (1.6-3.0) | 3.0 (1.1-4.9) | 0.474 |
| PSP | 72.6 (70.6-74.6) | 73.1 (67.6-78.5) | 0.878 | 72.1 (70.1-74.1) | 75.2 (68.9-81.6) | 0.351 | 73.3 (69.4-77.3) | 68.0 (62.1-73.8) | 0.151 |
| SDS | 6.4 (5.3-7.5) | 6.7 (2.3-11.2) | 0.891 | 5.3 (3.9-6.7) | 6.8 (2.2-11.4) | 0.549 | 7.3 (5.5-9.1) | 8.7 (3.1-14.2) | 0.656 |
| WHO-5 | 56.7 (53.7-59.7) | 58.8 (44.9-72.7) | 0.778 | 60.7 (56.3-65.2) | 64.6 (49.2-80.0) | 0.634 | 61.6 (56.0-67.1) | 52.9 (27.8-78.0) | 0.511 |
| Personal Control^b^ | 23.2 (22.5-23.9) | 22.2 (20.1-24.3) | 0.366 | 23.7 (22.9-24.5) | 22.3 (18.2-26.3) | 0.506 | 22.9 (21.9-24.0) | 21.7 (17.6-25.9) | 0.578 |
| Control/Manage Depression^c^ | 7.0 (6.7-7.2) | 6.2 (4.9-7.6) | 0.270 | 7.3 (6.9-7.6) | 5.8 (4.1-7.6) | 0.121 | 7.0 (6.4-7.6) | 5.7 (3.9-7.5) | 0.184 |
| Obtain Help from Community, Family, Friends^c^ | 7.1 (6.7-7.4) | 6.6 (4.9-8.3) | 0.598 | 7.4 (6.9-7.8) | 7.7 (5.9-9.6) | 0.706 | 7.1 (6.5-7.8) | 6.7 (4.5-8.9) | 0.720 |
| EQ-5D-3 L | 6.5 (6.2-6.7) | 6.8 (5.7-7.9) | 0.594 | 6.2 (5.9-6.5) | 6.7 (5.5-8.0) | 0.449 | 6.2 (5.8-6.6) | 6.7 (5.1-8.3) | 0.516 |
| PRISE^d^ | 14.3 (11.3-17.2) | 14.4 (6.0-22.9) | 0.971 | 13.8 (10.2-17.5) | 12.6 (0.0-29.3) | 0.882 | 19.4 (15.1-23.6) | 15.2 (0.0-32.6) | 0.652 |
| INSPIRE-S | 70.6 (66.1-75.1) | 66.4 (48.4-84.4) | 0.650 | 80.8 (75.7-85.9) | 73.0 (34.7-100) | 0.691 | 70.2 (63.4-77.0) | 63.5 (33.5-93.5) | 0.670 |
| INSPIRE-R | 83.6 (80.5-86.7) | 79.0 (65.3-92.7) | 0.518 | 89.7 (85.4-94.0) | 93.5 (70.5-100) | 0.758 | 84.1 (78.9-89.4) | 72.7 (52.1-93.4) | 0.299 |
| CSQ-8 | 26.0 (25.2-26.9) | 23.9 (16.9-30.9) | 0.542 | 28.0 (26.9-29.1) | 25.9 (14.0-37.8) | 0.724 | 26.3 (24.9-27.8) | 20.5 (8.6-32.4) | 0.339 |

Abbreviations: BDI-II: Beck Depression Inventory-II, BAI: Beck Anxiety Inventory, CC: Collaborative care, CSQ-8: Client Satisfaction Questionnaire, EQ-5D-3L: EuroQol Five Dimensions Questionnaire with Three Levels, GAF-F: Global Assessment of Functioning, INSPIRE-S: Recovery support from staff (Support), INSPIRE-R: Recovery support from staff (Relationship), PRISE: Patient Rated Inventory of Side Effects, PSP: Personal and Social Performance Scale, SCL-90-R: Symptom Checklist-90-Revised, SDS: Sheehan Disability Scale, TAU: Treatment-as-usual, WHO-5: World Health Organization-5 Well-Being Index.

Note: Means are estimated based on imputed data. In BDI-II, BAI, SCL-90-R, SDS, The Diagnostic Apathia Scale, and PRISE, lower scores are associated with a better outcome. In GAF, PSP, WHO-5, Personal control subscale from IPQ-R, Control/manage Depression subscale, Obtain Help from Community, Family, Friends subscale, EQ-5D-3L, CSQ-8, INSPIRE-S, and INSPIRE-R, higher scores are associated with a better outcome.

^a^ SCL-90-R was modified slightly as a reference period of two weeks was used instead of one week. ^b^ Subscale from the Illness Perception Questionnaire-Revised (IPQ-R). ^c^ Subscale from the Chronic Disease Self-Efficacy Scales. ^d^ Side effects are reported for the proportion of participants who used medication.
