# Supplementary material for: Collaborative care for depression and anxiety disorders: results and lessons learned from the Danish cluster-randomized Collabri trials
Source: BMC Fam Pract. 2020 Nov 18;21:234. doi: 10.1186/s12875-020-01299-3 (PMC7673096; doi:10.1186/s12875-020-01299-3)
Supplement: Supplementary file 4 — Additional file 4: Table A4. Questionnaire-based outcome means in the anxiety trials at 15-months’ follow-up. [file 12875_2020_1299_MOESM4_ESM.docx]

Table A4. Questionnaire-based outcome means in the anxiety trials at 15-months’ follow-up

|  | Generalized Anxiety Disorder | |  | Panic Disorder | |  | Social Anxiety Disorder | |  |
| --- | --- | --- | --- | --- | --- | --- | --- | --- | --- |
|  | CC (n=174) | TAU (n=19) |  | CC (n=124) | TAU (n=12) |  | CC (n=71) | TAU (n=6) |  |
|  | Mean (95% CI) | Mean (95% CI) | P | Mean (95% CI) | Mean (95% CI) | P | Mean (95% CI) | Mean (95% CI) | P |
| Primary outcome | | | | | | | | | |
| BAI | 11.8 (10.5-13.0) | 13.5 (9.3-17.6) | 0.436 | 10.0 (8.8-11.4) | 11.3 (7.3-15.3) | 0.568 | 11.0 (9.1-12.9) | 12.4 (6.0-18.8) | 0.677 |
| Secondary outcomes | | | | | | | | | |
| BDI-II | 9.8 (8.7-11.0) | 11.0 (5.7-16.3) | 0.675 | 7.5 (6.2-8.8) | 9.6 (6.2-13.0) | 0.259 | 10.0 (7.9-12.1) | 12.5 (4.5-20.4) | 0.562 |
| SCL-90-R^a^ | 50.3 (43.2-57.4) | 41.3 (8.2-74.5) | 0.607 | 40.7 (33.0-8.4) | 54.0 (31.3-76.6) | 0.280 | 51.8 (40.7-62.9) | 22.9 (0.0-80.8) | 0.341 |
| GAF | 75.9 (73.8-78.0) | 84.7 (69.8-99.6) | 0.252 | 74.0 (71.6-76.3) | 78.0 (68.0-87.9) | 0.441 | 73.4 (69.5-77.3) | 70.8 (53.5-88.0) | 0.769 |
| Explorative outcomes | | | | | | | | | |
| The Diagnostic Apathia Scale | 2.5 (2.0-2.9) | 2.1 (0.4- 3.7) | 0.670 | 1.7 (1.4- 2.1) | 1.8 (0.5- 3.1) | 0.932 | 2.0 (1.5-2.6) | 3.1 (0.4-5.7) | 0.444 |
| PSP | 75.9 (73.8-78.0) | 88.9 (65.6-100.0) | 0.274 | 74.9 (72.5-77.3) | 70.4 (45.0-95.7) | 0.725 | 74.0 (70.2-77.9) | 85.8 (52.3-100.0) | 0.492 |
| SDS | 5.4 (4.0-6.7) | 6.6 (3.3-10.0) | 0.483 | 5.6 (4.2-7.1) | 6.7 (2.9-10.5) | 0.602 | 5.4 (3.5-7.4) | 9.2 (3.1-15.3) | 0.247 |
| WHO-5 | 60.9 (57.0-64.9) | 57.8 (38.9-76.8) | 0.756 | 64.3 (59.8-68.8) | 58.9 (35.8-82.1) | 0.642 | 59.2 (53.0-65.5) | 26.1 (0- 81.2) | 0.243 |
| Personal Control^b^ | 19.7 (19.2-20.1) | 20.3 (19.1-21.5) | 0.298 | 20.2 (19.7-20.6) | 20.3 (18.8-21.7) | 0.916 | 20.0 (19.3-20.7) | 19.9 (17.7-22.1) | 0.933 |
| Control/Manage Depression^c^ | 7.1 (6.8-7.4) | 7.3 (6.2-8.3) | 0.773 | 7.5 (7.1-7.9) | 7.0 (5.9-8.0) | 0.361 | 6.7 (6.1-7.2) | 7.0 (5.5-8.5) | 0.696 |
| Obtain Help from Community, Family, Friends^c^ | 7.3 (7.0-7.2) | 7.1 (6.0-8.3) | 0.739 | 7.8 (7.4-8.1) | 7.8 (6.8-8.9) | 0.899 | 6.8 (6.3-7.4) | 6.9 (5.3-8.5) | 0.935 |
| EQ-5D-3 L | 0.8 (0.8-0.9) | 0.9 (0.8-0.9) | 0.292 | 0.9 (0.8-0.9) | 0.9 (0.8-1.0) | 0.824 | 0.8 (0.8-0.9) | 0.8 (0.7-0.9) | 0.887 |
| PRISE^d^ | 11.7 (9.1-14.4) | 14.9 (0.5-29.3) | 0.666 | 11.5 (8.0-14.9) | 12.8 (0.0-27.8) | 0.863 | 14.5 (10.1-18.9) | 20.0 (0.0-65.0) | 0.815 |

Abbreviations: BDI-II: Beck Depression Inventory-II, BAI: Beck Anxiety Inventory, CC: Collaborative care, CSQ-8: Client Satisfaction Questionnaire, EQ-5D-3L: EuroQol Five Dimensions Questionnaire with Three Levels, GAF-F: Global Assessment of Functioning, PRISE: Patient Rated Inventory of Side Effects, PSP: Personal and Social Performance Scale, SCL-90-R: Symptom Checklist-90-Revised, SDS: Sheehan Disability Scale, TAU: Treatment-as-usual, WHO-5: World Health Organization-5 Well-Being Index.

Note: Means are estimated based on imputed data. In BDI-II, BAI, SCL-90-R, SDS, The Diagnostic Apathia Scale, and PRISE, lower scores are associated with a better outcome. In GAF, PSP, WHO-5, Personal control subscale from IPQ-R, Control/manage Depression subscale, Obtain Help from Community, Family, Friends subscale, and EQ-5D-3L higher scores are associated with a better outcome.

^a^ SCL-90-R was modified slightly as a reference period of two weeks was used instead of one week. ^b^ Subscale from the Illness Perception Questionnaire-Revised (IPQ-R). ^c^ Subscale from the Chronic Disease Self-Efficacy Scales. ^d^ Side effects are reported for the proportion of participants who used medication.
