# Supplementary material for: Collaborative care for depression and anxiety disorders: results and lessons learned from the Danish cluster-randomized Collabri trials
Source: BMC Fam Pract. 2020 Nov 18;21:234. doi: 10.1186/s12875-020-01299-3 (PMC7673096; doi:10.1186/s12875-020-01299-3)
Supplement: Supplementary file 5 — Additional file 5: Table A5. Additional costs of collaborative care compared to treatment-as-usual. [file 12875_2020_1299_MOESM5_ESM.docx]

Table A5. Additional costs of collaborative care compared to treatment-as-usual

| Cost | EURO per person over the period of 6 months |
| --- | --- |
| Health insurance covered benefits | **-100.94** |
| Hospital care | -614.90 |
| Sick leave benefit^a^ | +341.88 |
| Unemployment benefit^a^ | -113.96 |
| Prescription drugs | +2.28 |
| Intervention costs | +1942.28 |
| Total | +1456.64 |

Note: -/+ indicates a lower/higher cost of collaborative care. Bold denotes statistically significant differences in cost between intervention and control group in register analysis (p-value less than 0.05).

Source: Own calculations based on intervention data and register data.

^a^ Estimated calculation from the maximum unemployment benefit/sick leave benefit rate.
