# Supplementary material for: Collaborative care for depression and anxiety disorders: results and lessons learned from the Danish cluster-randomized Collabri trials
Source: BMC Fam Pract. 2020 Nov 18;21:234. doi: 10.1186/s12875-020-01299-3 (PMC7673096; doi:10.1186/s12875-020-01299-3)
Supplement: Supplementary file 6 — Additional file 6: Table A6. Mean QALYs in the CC group and TAU group based on 6-months follow-up. [file 12875_2020_1299_MOESM6_ESM.docx]

Table A6. Mean QALYs in the CC group and TAU group based on 6-months follow-up

|  | CC (n=376) | TAU (n=57) | Difference in QALYs between CC and TAU |
| --- | --- | --- | --- |
| Adjusted QALY (95% CI) | 0.377 (0.371-0.384) | 0.352 (0.333-0.370) | 0.025 (P=0.006) |

Abbreviations: CC: Collaborative care, TAU: Treatment-as-usual.

Note: Analyses are based on complete answers to the EQ-5D-3L questionnaire. Means are adjusted for differences in the EQ-5D-3L score at baseline.

Source: Own calculations based on data from the Capital Region and Wittrup-Jensen et al. (1)

References:

1. Wittrup-Jensen KU, Lauridsen J, Pedersen KM, Gudex C. Generation of a Danish TTO value set for EQ-5D health states. Scand J Public Health. 2009;37(5):459–66.
