# Supplementary material for: Collaborative care for depression and anxiety disorders: results and lessons learned from the Danish cluster-randomized Collabri trials
Source: BMC Fam Pract. 2020 Nov 18;21:234. doi: 10.1186/s12875-020-01299-3 (PMC7673096; doi:10.1186/s12875-020-01299-3)
Supplement: Supplementary file 7 — Additional file 7. Consort 2010 checklist of information to include when reporting a randomized trial. [file 12875_2020_1299_MOESM7_ESM.doc]

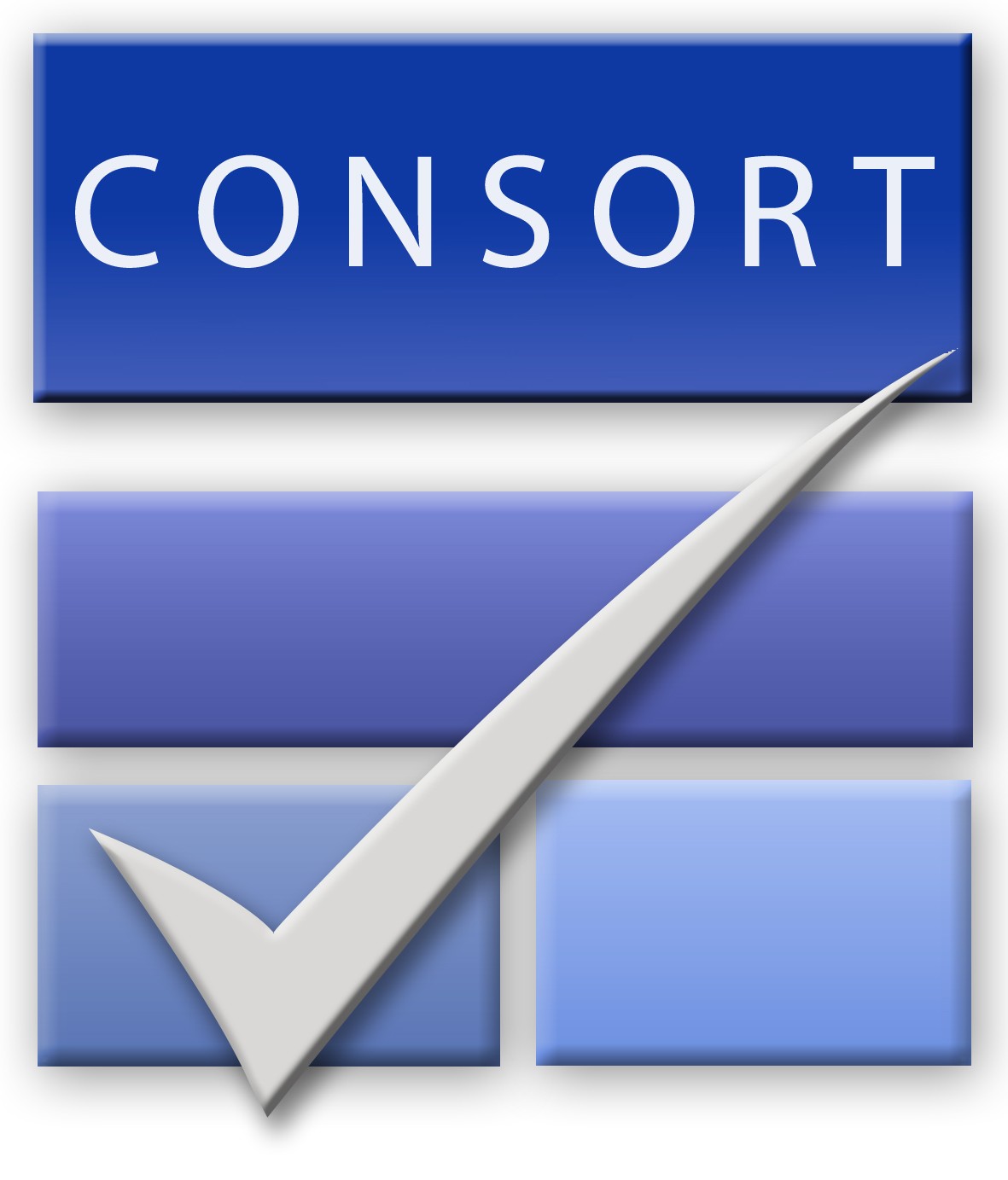
CONSORT 2010 checklist of information to include when reporting a randomised trial*

| Section/Topic | Item No | Checklist item | Reported on page No |
| --- | --- | --- | --- |
| Title and abstract | | | |
|  | 1a | Identification as a randomised trial in the title | Title |
|  | 1b | Structured summary of trial design, methods, results, and conclusions (for specific guidance see CONSORT for abstracts) | Abstract |
| Introduction | | | |
| Background and objectives | 2a | Scientific background and explanation of rationale | Background |
|  | 2b | Specific objectives or hypotheses | Background: Paragraph 2  Methods: Section under subheading *Design* |
| Methods | | | |
| Trial design | 3a | Description of trial design (such as parallel, factorial) including allocation ratio | Methods: Section under subheading *Design*  Methods: Section under subheading *Recruitment of general practitioners and randomization* paragraph 2 |
|  | 3b | Important changes to methods after trial commencement (such as eligibility criteria), with reasons | Methods: Section under subheading *Statistical analyses*, paragraph 2 |
| Participants | 4a | Eligibility criteria for participants | Methods: Section under subheading *Recruitment of general practitioners and randomization,* paragraph 1  Methods: Section under subheading *Population* |
|  | 4b | Settings and locations where the data were collected | Methods: Section under subheading *Recruitment of general practitioners and randomization*, paragraph 1  Methods: Section under subheading *Interventions, The Collabri model of collaborative care*, paragraph 2 |
| Interventions | 5 | The interventions for each group with sufficient details to allow replication, including how and when they were actually administered | Methods: Section under subheading *Interventions* |
| Outcomes | 6a | Completely defined pre-specified primary and secondary outcome measures, including how and when they were assessed | Methods: Section under subheading *Outcomes and data*, paragraph 1.  Table 1: *Primary outcomes* and *Secondary outcomes* |
|  | 6b | Any changes to trial outcomes after the trial commenced, with reasons | Methods: Section under subheading *Outcomes and data*, paragraph 2 |
| Sample size | 7a | How sample size was determined | Methods: Section under subheading *Sample size calculations* |
|  | 7b | When applicable, explanation of any interim analyses and stopping guidelines | N/A |
| Randomisation: |  |  |  |
| Sequence generation | 8a | Method used to generate the random allocation sequence | Methods: Section under subheading *Recruitment of general practitioners and randomization*, paragraph 1 |
|  | 8b | Type of randomisation; details of any restriction (such as blocking and block size) | Methods: Section under subheading *Recruitment of general practitioners and randomization*, paragraph 1 |
| Allocation concealment mechanism | 9 | Mechanism used to implement the random allocation sequence (such as sequentially numbered containers), describing any steps taken to conceal the sequence until interventions were assigned | Methods: Section under subheading *Recruitment of general practitioners and randomization*, paragraph 1 |
| Implementation | 10 | Who generated the random allocation sequence, who enrolled participants, and who assigned participants to interventions | Methods: Section under subheading *Recruitment of general practitioners and randomization*, paragraph 1  Methods: Section under subheading *Recruitment of participants* |
| Blinding | 11a | If done, who was blinded after assignment to interventions (for example, participants, care providers, those assessing outcomes) and how | Methods: Section under subheading *Blinding* |
|  | 11b | If relevant, description of the similarity of interventions | N/A |
| Statistical methods | 12a | Statistical methods used to compare groups for primary and secondary outcomes | Methods: Section under subheading *Statistical analyses*, paragraph 1 |
|  | 12b | Methods for additional analyses, such as subgroup analyses and adjusted analyses | Methods: Section under subheading *Statistical analyses*  Methods: Section under *Health Economic evaluation*, paragraph 2 and 3 |
| Results | | | |
| Participant flow (a diagram is strongly recommended) | 13a | For each group, the numbers of participants who were randomly assigned, received intended treatment, and were analysed for the primary outcome | Figure 1  Results: Section under subheading *Characteristics of participating patients* |
|  | 13b | For each group, losses and exclusions after randomisation, together with reasons | Figure 1 |
| Recruitment | 14a | Dates defining the periods of recruitment and follow-up | Results: Section under subheading *Characteristics of participating general practitioners,* first line  Results: Section under subheading *Characteristics of participating patients*, first line  Methods: Section under subheading *Outcomes and data*, paragraph 1 |
|  | 14b | Why the trial ended or was stopped | Background: Paragraph 2 |
| Baseline data | 15 | A table showing baseline demographic and clinical characteristics for each group | Table 2 |
| Numbers analysed | 16 | For each group, number of participants (denominator) included in each analysis and whether the analysis was by original assigned groups | Methods: Section under subheading *Statistical analyses*, paragraph 1  Results: Section under subheading *Characteristics of participating patients* |
| Outcomes and estimation | 17a | For each primary and secondary outcome, results for each group, and the estimated effect size and its precision (such as 95% confidence interval) | Table 5 and 8 |
|  | 17b | For binary outcomes, presentation of both absolute and relative effect sizes is recommended | Table 7 (sick leave benefits) |
| Ancillary analyses | 18 | Results of any other analyses performed, including subgroup analyses and adjusted analyses, distinguishing pre-specified from exploratory | Results: Section under subheading *The depression trial*, paragraph 2  Results: Section under subheading *The pooled anxiety trial*, paragraph 2  Results: Section under subheading *Health economic evaluation* |
| Harms | 19 | All important harms or unintended effects in each group (for specific guidance see CONSORT for harms) | Results: Section under subheading *Harms* |
| Discussion | | | |
| Limitations | 20 | Trial limitations, addressing sources of potential bias, imprecision, and, if relevant, multiplicity of analyses | Discussion: Section under subheading *Lessons learned, strengths and limitations* |
| Generalisability | 21 | Generalisability (external validity, applicability) of the trial findings | Discussion: Section under subheading *Lessons learned, strengths and limitations,* paragraph 4 |
| Interpretation | 22 | Interpretation consistent with results, balancing benefits and harms, and considering other relevant evidence | Conclusion |
| Other information | | |  |
| Registration | 23 | Registration number and name of trial registry | Section under subheading *Trial Registration* |
| Protocol | 24 | Where the full trial protocol can be accessed, if available | N/A |
| Funding | 25 | Sources of funding and other support (such as supply of drugs), role of funders | Declarations: Section under subheading *Funding* |

*We strongly recommend reading this statement in conjunction with the CONSORT 2010 Explanation and Elaboration for important clarifications on all the items. If relevant, we also recommend reading CONSORT extensions for cluster randomised trials, non-inferiority and equivalence trials, non-pharmacological treatments, herbal interventions, and pragmatic trials. Additional extensions are forthcoming: for those and for up to date references relevant to this checklist, see [www.consort-statement.org](http://www.consort-statement.org/).
